# Standardized tumor volume: an independent prognostic factor in advanced nasopharyngeal carcinoma

**DOI:** 10.18632/oncotarget.20313

**Published:** 2017-08-17

**Authors:** Ting Liu, Jun Lv, Yutao Qin

**Affiliations:** ^1^ Department of Radiotherapy, The First Affiliated Hospital of Guangxi Medical University, Nanning 530021, Guangxi, China

**Keywords:** nasopharyngeal carcinoma, standardized tumor volume, tumor burden, intensity-modulated radiotherapy, prognosis

## Abstract

The study evaluated the prognostic effect of standardized tumor volume in patients with advanced nasopharyngeal carcinoma (NPC) treated with concurrent chemoradiotherapy. Between Jan 1, 2009 and December 30, 2012, 143 patients diagnosed with NPC in UICC stage III–IVb by histopathology were enrolled in the study. These patients underwent intensity-modulated radiotherapy combined with concurrent chemotherapy. The three-dimensional images of tumor volume were reconstructed automatically by the treatment planning system. SGTVnx was calculated based on GTVnx/person’s volume. SGTVnd was calculated based on GTVnd/person’s volume. SGTVnx was significantly associated with the 5-year overall survival (OS), disease-free survival (DFS), DMFS, and LRFS rates in univariate and multivariate analyses. Although SGTVnd was associated with the 5-year OS, DFS, and DMFS rates, it was not an independent prognostic factor for LRFS. In receiver operating characteristic (ROC) curve analysis, 1.091 and 0.273 were determined as the cut-off points for SGTVnx and SGTVnd, respectively. The 5-year OS, DFS, DMFS, and LRFS rates for patients with a SGTVnx > 1.091 *vs.* SGTVnx ≤ 1.091 was 65.4% *vs.* 93.4% (*P <* 0.001), 65.2% *vs.* 94.8% (*P <* 0.001), 71.4% *vs.* 97.4% (*P <* 0.001), and 84.8% *vs.* 97.3% (*P =* 0.003), respectively, for SGTVnd > 0.273 *vs.* SGTVnd ≤ 0.273 was 70.3% *vs.* 96.5% (*P <* 0.001), 70.1% *vs.* 94.8% (*P <* 0.001), 77.5% *vs.* 98.2% (*P <* 0.001), and 88.5% *vs.* 96.6% (*P =* 0.049), respectively. UICC stage grouping, T classification, N classification, and sex were not found to be independent prognostic factors for NPC. Standardized tumor volume was an independent prognostic factor for NPC that might improve the current NPC TNM classification system and provide new clinical evidence for personalized treatment strategies.

## INTRODUCTION

Nasopharyngeal carcinoma (NPC) has a unique, unbalanced, endemic distribution: 86,700 new cases of NPC and 50,800 deaths in 2012 with the highest incidences reported in Southeast Asia, Micronesia, Polynesia, Eastern Asia, and northern Africa [1–3]. Also, high rate of incidences are observed in several provinces in South-Eastern China, with an age-standardized incidence rate of 20–50/100,000 males [2]. Unlike other head and neck cancers, radiotherapy is the uppermost treatment modality for NPC owing to its anatomical location and radiation sensitivity [4].

Since tumor volume can be calculated by the planning system, it can be considered a potential prognostic factor [5, 6]. In recent years, several studies have confirmed a significant correlation between tumor volume and survival rate [7–9]. Although most of the clinicians are in agreement, with this opinion, it continues to remain a major concern. Different studies postulate different cut-offs for tumor volume; for instance, Sze et al. showed that the cutoff point of tumor volume was 15 mL, Guo et al. indicated that 19 mL could be an adequate independent risk factor, whereas another 2 studies reported that the cutoff point for GTVp (gross tumor volume of the primary) was larger than that described in the other studies, 50 and 60 mL [7].

The current TNM classification system of NPC is the most widespread application system worldwide, as well as the most significant prognostic factor for NPC; however, it was formulated in the epoch of conventional two-dimensional radiotherapy. Thus, the tumor volume was not incorporated into the system, which might be attributed to the lack of a widely accepted, effective, and available standard approach [10, 11].

Therefore, in the current study, we elucidated the prognostic value of tumor volume and proposed that the standardized tumor volume is based on the individual tumor burden differences in patients. This new theory was aimed to satisfactorily predict the survival, thereby establishing a wide acceptance as an effective and available standard.

## RESULTS

### Patient demographics

Between Jan 1, 2009 and December 30, 2012, 143 patients with advanced NPC were enrolled in the present study. The ratio of male to female was 3:1. A total of 55 (38.5%) patients presented stage III, and 88 (61.5%) presented stage IVa and IVb. All the patients were WHO II with respect to histological classification, and concurrent chemoradiotherapy was administered in all patients. The median follow-up time was 62.94 (range: 2–105) months. The clinical characteristics of the 143 NPC patients are listed in Table 1.

**Table 1:** Patient characteristics

Characteristic	*n* (%)
Age (years)	46.15 ± 11.43
> 50	50 (35.0)
≤ 50	93 (65.0)
Sex	
Male	108 (75.5)
Female	35 (24.5)
UICC stage	
T1/T2/T3/T4	3(2.1)/12(8.4)/44(30.8)/84(58.7)
N0/N1/N2/N3	13 (9.1)/60(42.0)/64(44.8)/6(4.2)
III/IV	55(38.5)/88(61.5)
Histology	
WHO II	143(100.0)
Follow-up time(months)	62.94 ± 22.46

### Optimal threshold for tumor volume

A receiver operating characteristic (ROC) curve analysis was performed to determine the cut-offs for the correlation between GTVnx, GTVnd, and OS, which were 43.48 mL and 15.005 mL, respectively. The cut-off points for the correlation between SGTVnx/SGTVnd and OS were1.091 and 0.273, respectively.

### Overall survival (OS)

For all the patients, the 1, 3, 5, and 8-year OS was 95.8, 85.3, 80.9, and 78.4%, respectively. 29 patients succumbed to mortality: 12 cases of distant metastasis, 4 locoregional recurrence, 4 of both distant metastasis and recurrence, 1 case of hemorrhage in nasopharynx, 1 case of traffic accident, 1 case of a second primary malignancy, 1 case of heart disease, 1 case of cerebral infarction, and 4 of unknown causes.

The ROC curve analysis determined the cut-offs for the correlation between GTVnx/GTVnd and OS, which were 43.48 mL and 15.005 mL, respectively. The 5-year OS rates in patients with a GTVnx > 43.48 mL *vs*. GTVnx ≤ 43.48 mL was 67.9 *vs*. 96.9% (*P <* 0.001), GTVnd > 15.005 mL *vs*. GTVnd ≤ 15.005 mL was 73.5 *vs*. 91.4% (*P <* 0.001).

**Figure 1:**
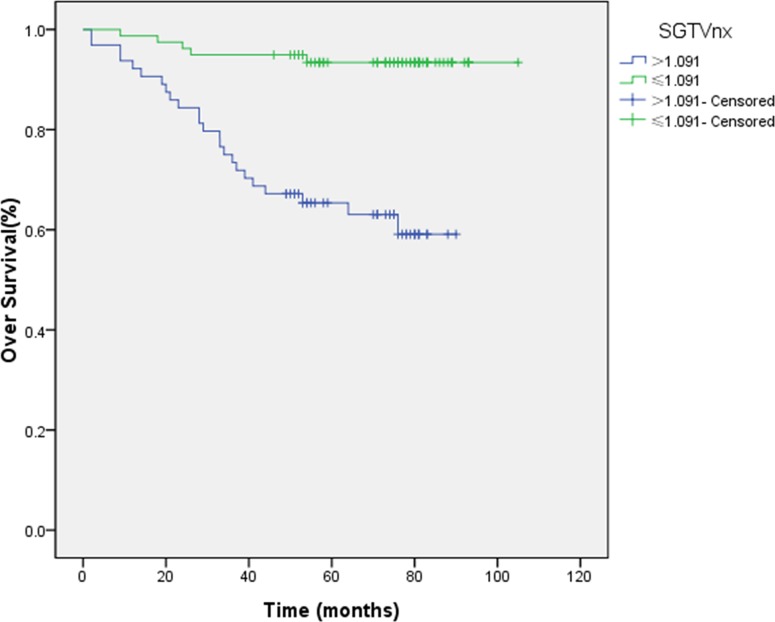
Effect of standardized primary gross volume for nasopharynx (SGTVnx) on overall survival

**Figure 2:**
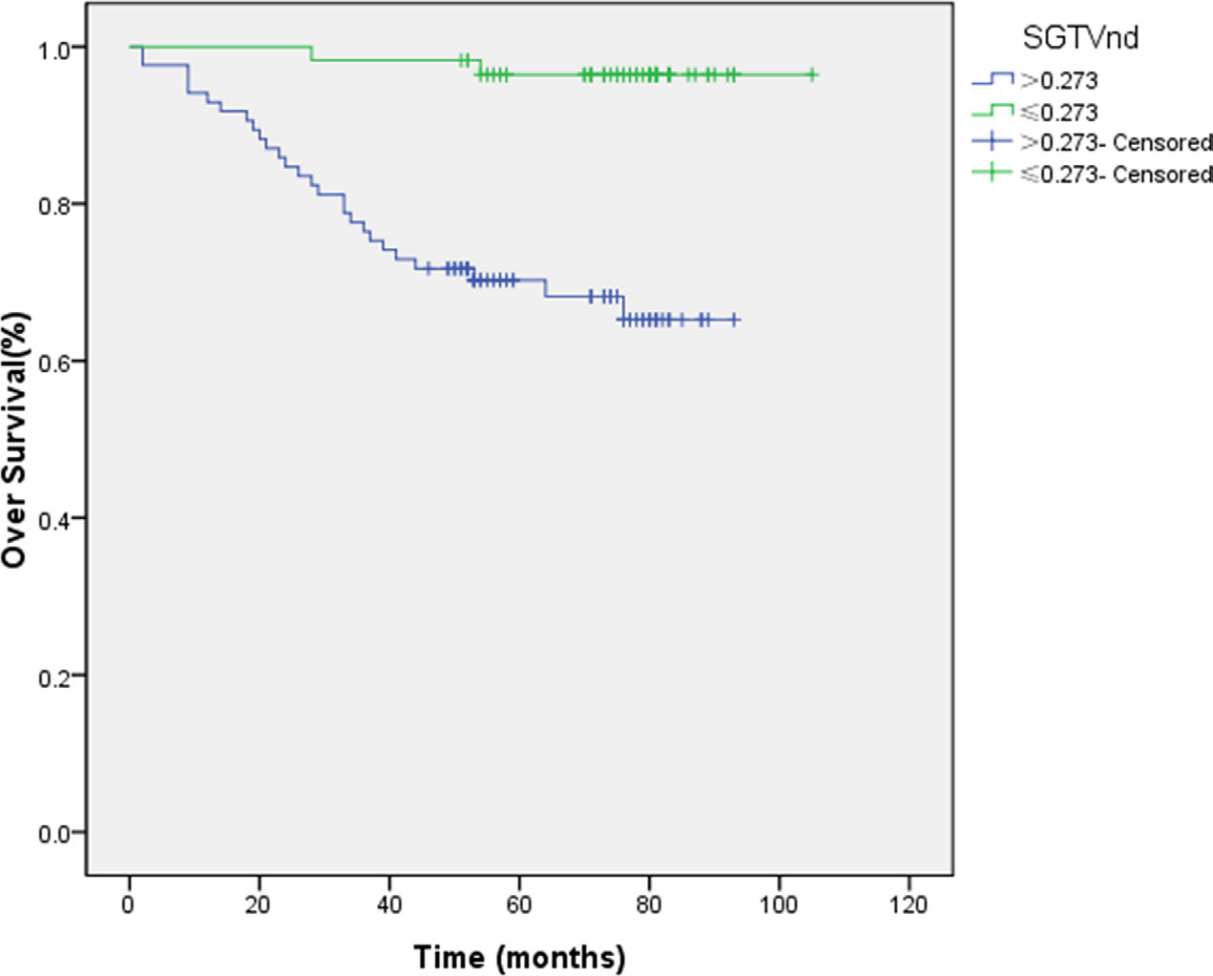
Effect of standardized primary gross volume for lymph nodes (SGTVnd) on overall survival

In order to consider the individual tumor burden, we proposed a new theory of standardized tumor volume. SGTVnx and SGTVnd were calculated by GTVnx/person’s volume. We also used the ROC curve analysis to determine the cut-off points which were 1.091 and 0.273, respectively. The 5-year OS rates for patients with a SGTVnx > 1.091 *vs*. SGTVnx ≤ 1.091 was 65.4 *vs*. 93.4% (*P <* 0.001, Figure 1), for SGTVnd > 0.273 *vs*. SGTVnd ≤ 0.273 was 70.3 *vs*. 96.5% (*P <* 0.001, Figure 2).

The Kaplan–Meier method was utilized in the univariate analysis, and the difference was analyzed using a two-sided log-rank test. The result showed that GTVnx (*P <* 0.001), GTVnd (*P =* 0.003), SGTVnx (*P <* 0.001), SGTVnd (*P <* 0.001), UICC stage grouping (*P <* 0.001), T classification (*P =* 0.001), age (*P =* 0.002) significantly correlated with the 5-year OS rates, whereas a similar phenomenon was not established with respect to the N classification and gender (*P >* 0.05, Table 2).

**Table 2:** Impact of prognostic factors on treatment according to univariate analysis (log-rank test)

Characteristics		OS	Log-Rank test	*P*	DFS	Log-Rank test	*P*	DMFS	Log-Rank test	*P*	LRFS	Log-Rank test	*P*
Sex													
Male	108	79.3%	0.237	0.627	81.8%	.028	0.867	84.7%	0.987	0.320	92.6%	0.828	0.363
Female	35	85.7%			82.1%			90.8%			90.8%		
Age (years)													
> 50	50	70.0%	9.884	**0.002**	73.6%	5.030	**0.025**	77.7%	4.544	**0.033**	92.6%	0.240	0.624
≤ 50	93	86.7%			85.9%			90.2%			92.1%		
UICC stage													
IV	88	72.1%	12.327	**< 0.001**	75.1%	9.082	**0.003**	79.7%	8.617	**0.003**	89.4%	2.931	0.087
III	55	94.5%			92.6%			96.3%			96.2%		
T classification													
T4	84	71.8%	11.648	**0.001**	75.1%	8.454	**0.004**	79.9%	7.405	**0.007**	89.0%	3.531	0.060
T1–3	59	93.2%			91.4%			94.8%			96.4%		
N classification													
N2–3	70	78.6%	0.202	0.653	79.1%	.326	0.568	83.6%	0.511	0.475	93.3%	0.458	0.498
N0–1	73	83.0%			84.6%			88.7%			91.1%		
GTVnx													
> 43.48 mL	79	67.9%	21.015	**< 0.001**	69.3%	20.807	**< 0.001**	75.8%	15.966	**< 0.001**	86.3%	8.150	**0.004**
≤ 43.48 mL	64	96.9%			96.8%			98.4%			98.4%		
GTVnd													
> 15.005 mL	84	73.5%	8.909	**0.003**	74.0%	10.614	**0.001**	78.9%	9.845	**0.002**	88.8%	3.499	0.061
≤ 15.005	59	91.4%			93.0%			96.5%			96.4%		
SGTVnx													
> 1.091	64	65.4%	21.765	**< 0.001**	65.2%	25.281	**< 0.001**	71.4%	21.245	**< 0.001**	84.8%	8.999	**0.003**
≤ 1.091	79	93.4%			94.8%			97.4%			97.3%		
SGTVnd													
> 0.273	85	70.3%	17.853	**< 0.001**	70.1%	14.046	**< 0.001**	77.5%	13.355	**< 0.001**	88.5%	3.892	**0.049**
≤ 0.273	58	96.5%			94.8%			98.2%			96.6%		

Cox regression was calculated by the multivariate analysis. The multivariate analysis showed that GTVnx (hazard ratio (HR) 0.128; *P =* 0.006), GTVnd (HR 0.282; *P =* 0.011), SGTVnx (HR 0.335; *P =* 0.037), SGTVnd (HR 0.106; *P =* 0.003), and age also significantly correlated with the 5-year OS, while UICC stage grouping and T classification were not correlated with the OS (*P >* 0.05, Tables 3–4).

**Table 3:** Multivariate analysis of prognostic factors

	OS				DFS				DMFS				LRFS			
Variable	HR	95% CI		*P*	HR	95% CI		*P*	HR	95% CI		*P*	HR	95% CI		*P*
Age	0.304	0.143	0.644	0.002	0.410	0.190	0.886	0.023	0.382	0.155	0.939	0.036				
UICC stage	0.155	0.018	1.373	0.094	0.233	0.031	1.767	0.159	0.145	0.014	1.491	0.104				
T classification	1.803	0.258	12.576	0.552	1.619	0.252	10.423	0.612	1.833	0.256	13.134	0.547				
GTVnx	0.128	0.030	0.558	0.006	0.119	0.027	0.519	0.005	0.092	0.012	0.706	0.022	0.091	0.012	0.713	0.023
GTVnd	0.282	0.106	0.751	0.011	0.234	0.080	0.686	0.008	0.158	0.036	0.690	0.014				

**Table 4:** Multivariate analysis of prognostic factors

	OS				DFS				DMFS				LRFS			
Variable	HR	95% CI		*P*	HR	95% CI		*P*	HR	95% CI		*P*	HR	95% CI		*P*
Age	0.295	0.139	0.624	0.001	0.423	0.195	0.917	0.029	0.400	0.162	0.985	0.046				
UICC stage	0.183	0.021	1.573	0.122	0.261	0.035	1.925	0.188	0.172	0.017	1.740	0.136				
T classification	1.542	0.222	10.695	0.661	1.607	0.253	10.196	0.615	1.805	0.256	12.739	0.553				
SGTVnx	0.335	0.120	0.938	0.037	0.204	0.066	0.634	0.006	0.162	0.036	0.742	0.019	0.176	0.036	0.866	0.033
SGTVnd	0.106	0.025	0.459	0.003	0.196	0.057	0.677	0.010	0.095	0.012	0.727	0.023	0.425	0.086	2.094	.293

### Disease-free survival (DFS)

For all patients, the 1, 3, 5, and 8-year DFS was 95.1, 84.1, 81.9, and 79.2%, respectively. 12 patients presented distant metastasis (3 cases of lung, 2 bone, 2 liver, 1 adrenal gland, and 4 multiple site metastasis), 4 patients exhibited locoregional recurrence, and 4 suffered from both distant metastasis and recurrence.

The ROC curve analysis determined that the cut-off for the correlation between GTVnx/GTVnd and 5-year OS was 43.48 mL and 15.005 mL, respectively. Thus, we also selected these points for classifying the patients into high and low groups to evaluate the DFS. The 5-year DFS rates for patients with a GTVnx > 43.48 mL *vs*. GTVnx ≤ 43.48 mL was 69.3 *vs*. 96.8% (*P <* 0.001), GTVnd > 15.005 mL *vs*. GTVnd ≤ 15.005 mL was 74.0 *vs*. 93.0% (*P =* 0.001).

**Figure 3:**
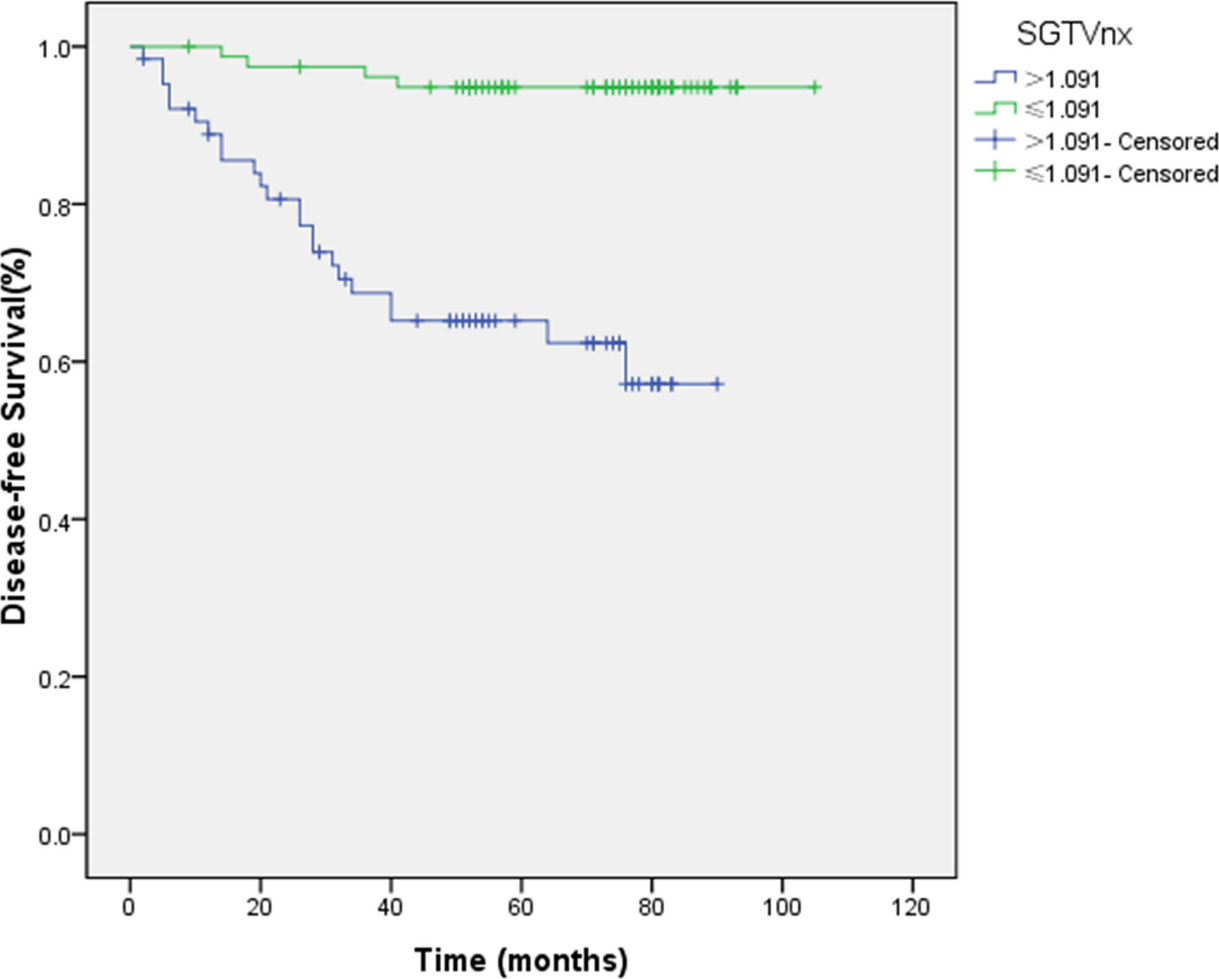
Effect of standardized primary gross volume for nasopharynx (SGTVnx) on disease-free survival

**Figure 4:**
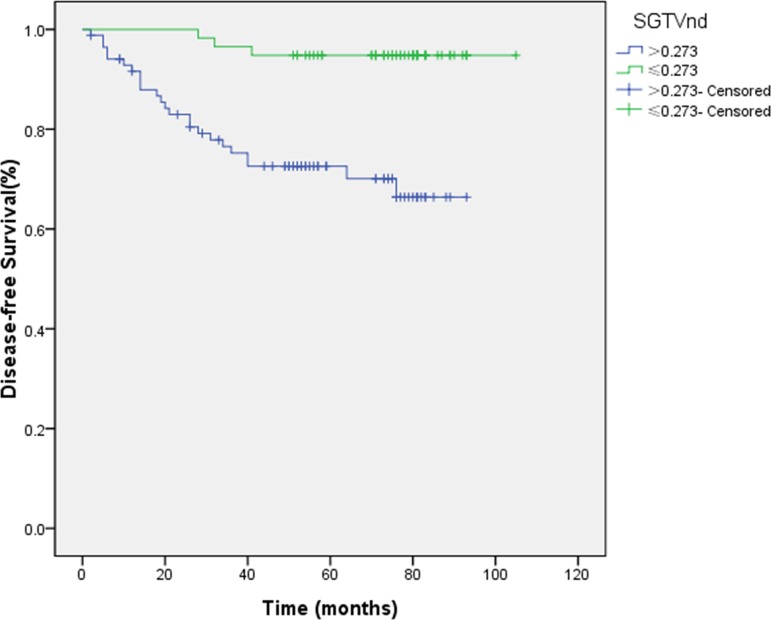
Effect of standardized primary gross volume for lymph nodes (SGTVnd) on disease-free survival

Similarly, the ROC curve analysis determined the cut-off points for the correlation between SGTVnx, SGTVnd and 5-yearOS, which was 1.091 and 0.273, respectively, and thus, these were selected for the evaluation of 5-year DFS. The 5-year DFS rates for patients with a SGTVnx > 1.091 *vs*. SGTVnx2 ≤ 1.091 was 65.2 *vs*. 94.8% (*P <* 0.001, Figure 3), SGTVnd > 0.273*vs*. SGTVnd ≤ 0.273 was 70.1 *vs*. 94.8% (*P <* 0.001, Figure 4). The Kaplan–Meier method was used in the univariate analysis. The result showed that GTVnx (*P <* 0.001), GTVnd (*P =* 0.001), SGTVnx (*P <* 0.001), SGTVnd (*P <* 0.001), UICC stage grouping (*P =* 0.003),T classification (*P =* 0.004), and age (*P =* 0.025) significantly correlated with the 5-year DFS rates; on the other hand, the N classification and sex of the patients did not correlate (*P >* 0.05, Table 2).

Furthermore, the multivariate analysis showed that GTVnx (HR 0.119; *P =* 0.005), GTVnd (HR 0.234; *P =* 0.008), SGTVnx (HR 0.204; *P =* 0.006), SGTVnd (HR 0.196; *P =* 0.010), and age also significantly correlated with the 5-year DFS, while UICC stage grouping and T classification were not associated with the phenomenon (*P >* 0.05, Tables 3–4).

### Distant metastasis-free survival (DMFS)

The rate of 1, 3, 5, and 8-year DMFS was 98.6, 91.2, 87.8, and 75.6%, respectively. Twelve patients presented distant metastasis (3 cases of lung, 2 bone, 2 liver, 1 adrenal gland, and 4 multiple site metastasis) and 4 cases of both distant metastasis and recurrence.

We selected 43.48 and 15.005 mL as the cut-off points for classifying all patients into high and low groups for DMFS. The 5-year DMFS rates for patients with a GTVnx > 43.48 mL *vs*. GTVnx ≤ 43.48 mL was 75.8 *vs*. 98.4% (*P <* 0.001), GTVnd > 15.005 mL *vs*. GTVnd ≤ 15.005 mL was 78.9 *vs*. 96.5% (*P =* 0.002).

**Figure 5:**
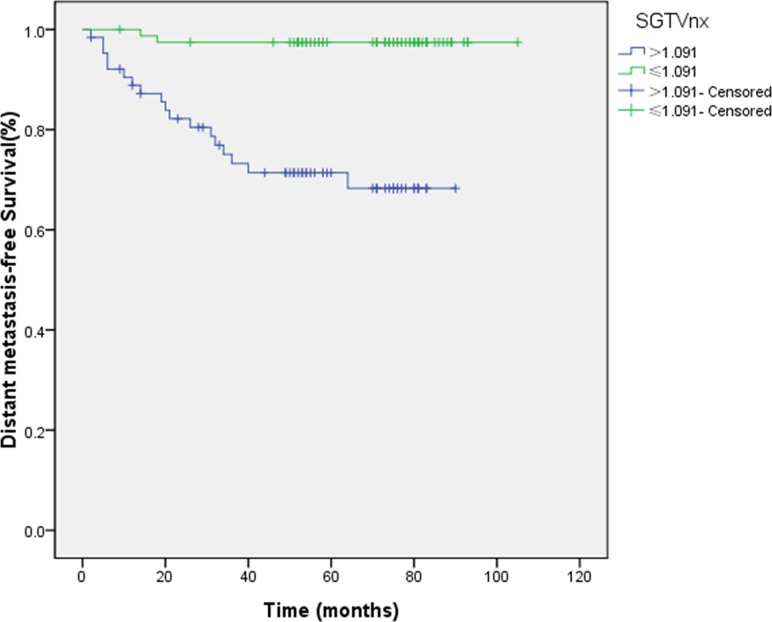
Effect of standardized primary gross volume for nasopharynx (SGTVnx) on distant metastasis-free survival

**Figure 6:**
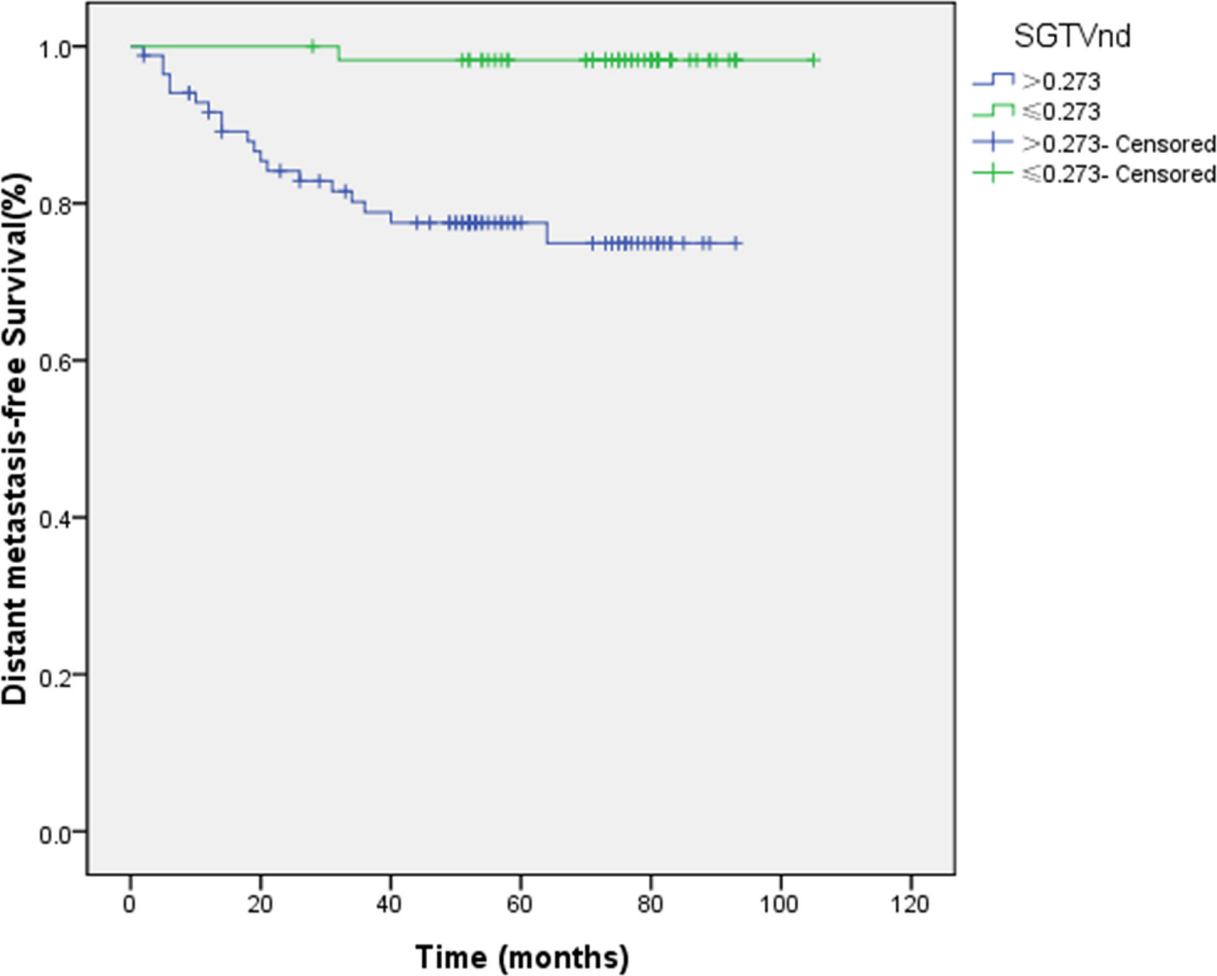
Effect of standardized primary gross volume for lymph nodes (SGTVnd) on distant metastasis-free survival

Similar to the above results, 1.091 and 0.273 were the cut-off points for the evaluation of DMFS. The 5-year DMFS rates for patients with SGTVnx > 1.091 *vs*. SGTVnx ≤ 1.091 was 71.4 *vs*. 97.4% (*P <* 0.001, Figure 5),SGTVnd > 0.273 *vs*. SGTVnd ≤ 0.273 was 77.5 *vs*. 98.2% (*P <* 0.001, Figure 6). The Kaplan–Meier method revealed that GTVnx (*P <* 0.001), GTVnd (*P =* 0.002), SGTVnx (*P <* 0.001), SGTVnd (*P <* 0.001), UICC stage grouping (*P =* 0.003), T classification (*P =* 0.007), and age (*P =* 0.033) significantly correlated with the 5-year DMFS rates, whereas N classification and sex did not correlate (*P >* 0.05, Table 2).

The multivariate analysis showed that GTVnx (HR 0.092; *P =* 0.022), GTVnd (HR 0.158; *P =* 0.008), SGTVnx (HR, 0.162; *P =* 0.014), SGTVnd (HR, 0.095; *P =* 0.023), and age also significantly correlated with the 5-year DMFS, while UICC stage grouping and T classification did not associate with the phenomenon (*P >* 0.05, Tables 3–4).

### Local relapse-free survival (LRFS)

The 1, 3, 5, and 8-year LRFS was 100, 95.4, 92.2, and 90.5%, respectively. Four patients exhibited locoregional recurrence, while 4 patients had cases of both distant metastasis and recurrence.

We selected 43.48 and 15.005 mL, as described above, to serve as the cut-off points for LRFS. The 5-year LRFS rates for patients with a GTVnx > 43.48 mL *vs*. GTVnx ≤ 43.48 mL was 86.3 *vs*. 98.4% (*P =* 0.004), GTVnd > 15.005 mL *vs*. GTVnd ≤ 15.005 mL was 88.8 *vs*. 96.4% (*P =* 0.061).

**Figure 7:**
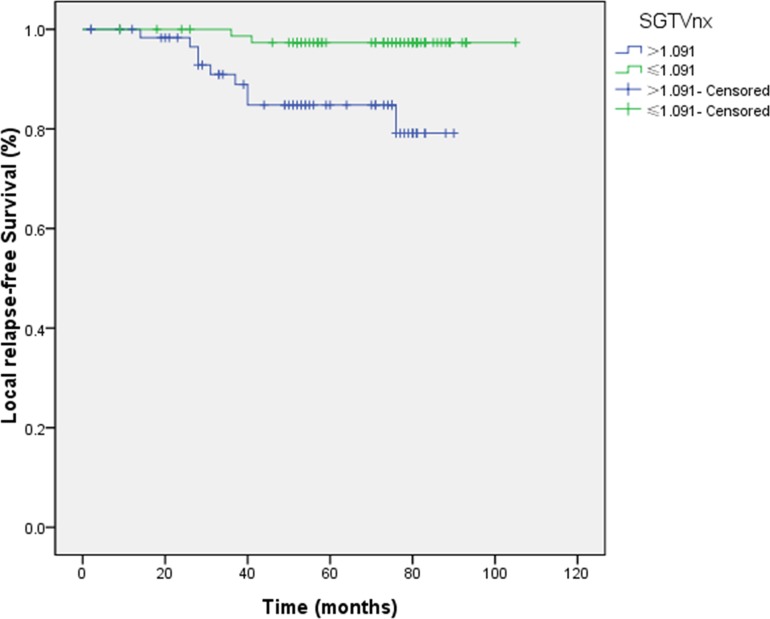
Effect of standardized primary gross volume for nasopharynx (SGTVnx) on local relapse-free survival

**Figure 8:**
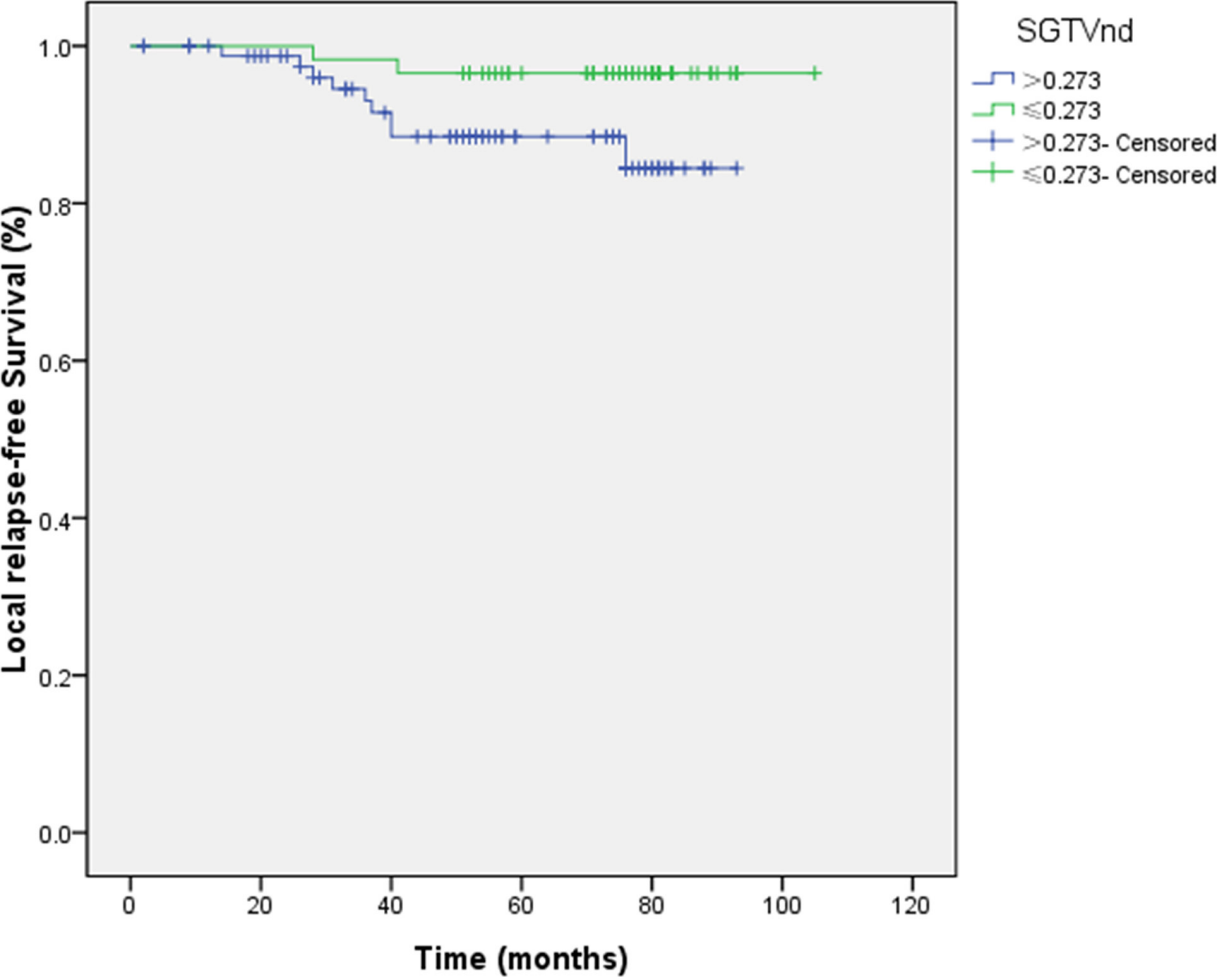
Effect of standardized primary gross volume for lymph nodes (SGTVnd) on local relapse-free survival

1.091 and 0.273 were the cut-off values for LRFS. The 5-year LRFS rates for patients with SGTVnx > 1.091 *vs*. SGTVnx ≤ 1.091 was 84.8 *vs*. 97.3% (*P =* 0.003, Figure 7), SGTVnd > 0.273 *vs*. SGTVnd ≤ 0.273 was 88.5 *vs*. 96.6% (*P =* 0.049, Figure 8).

The univariate analysis showed that GTVnx (*P =* 0.004), SGTVnx (*P =* 0.003), SGTVnd (*P =* 0.049) significantly correlated with The 5-year LRFS rates, whereas GTVnd, UICC stage grouping, T and N classifications, sex, and age did not associate with the survival (*P* > 0.05, Table 2). Subsequently, the multivariate analysis showed that GTVnx (HR 0.091; *P =* 0.023) and SGTVnx (HR 0.176; *P =* 0.033) also significantly correlated with the OS (Tables 3–4).

## DISCUSSION

Currently, IMRT has replaced the conventional two-dimensional radiotherapy as the pivotal radiotherapy technique [11]. Several studies have proposed novel theories that can predict the therapeutic response and the prognosis in order to consummate the existing UICC staging system in IMRT [10, 11]. Thus, selecting the optimal treatment plan for the patient is essential.

Although several factors affect prognosis, tumor volume is one of the critical prognostic factors. The blood supply to the center of a large tumor is usually insufficient, leading to a favorable microenvironment for the rapid proliferation of hypoxic and G0 cells, thereby rendering a low sensitivity to radiotherapy [12]. Large-sized tumors are connected with the increasing number of clonogenic tumor cells and other increasing unfavorable radiobiological factors. Therefore, a high radiation dose is obligatory for achieving satisfactory therapeutic effects [13, 14]. Tumor volume was confirmed as a valuable independent prognostic factor in several malignant tumors; it has been incorporated for staging and predicting prognosis in lung carcinoma, breast cancer, and other malignancies.

However, the tumor volume is not included in the existing UICC staging system in NPC [10] The value of tumor volume in predicting prognosis in patients with NPC has been investigated previously [15, 16]. Sze et al. reported that local failure-free survival, PFS, and OS were higher in patients with a tumor volume < 15 mL as compared to those with > 15 mL [17]. Guo et al. indicated that patients with a tumor volume > 19 mL could have a better prognosis [18]. Another two studies reported that the cutoff point for GTVp was large: 50 and 60 mL, respectively [19, 20]. We found that the tumor volume cut-off point was 33 mL in another multicenter study in our unit [7].

The current study demonstrated tumor volume as a major prognostic factor for NPC. The patients were assigned to 2 groups:

GTVnx > 43.48 mL and GTVnx ≤ 43.48 mL. The Kaplan–Meier method indicated that the 5-year OS rate for patients with smaller GTVnx was significantly higher than the patients with larger GTVnx; also DFS, LRFS, and DMFS were higher.

In order to distinguish the impact between primary tumors volume of nasopharynx with cervical metastatic lymph nodes volume [21], we categorized the patients into 2 groups according to GTVnd: GTVnd > 15.005 mL *vs*. GTVnd ≤ 15.005 mL. The survival curves indicated that the patients with smaller GTVnd had a better prognosis than those with larger GTVnd.

In our study, GTVnx and GTVnd were ascertained as independent prognostic factors for the 5-year OS, DFS, and DMFS by both univariate and multivariate analyses. GTVnx, but not GTVnd, was also the independent prognostic factor for LRFS. This consequence that may be caused by insufficient positive events may lead to bias. On the other hand, with the application of IMRT, patients with NPC are regulated with respect to the local recurrence that is closely associated with local invasion and GTVnx instead of GTVnd. Herein, we also demonstrated that UICC stage grouping, T and N classifications, and sex are not the independent prognostic factors for NPC. This feature indicate a limitation of the current UICC classification system.

We proposed that tumor volume is a superior prognostic factor for NPC than the existing UICC staging system in NPC. However, the cut-off of the tumor volume is different in different studies that do not allow its wide clinical application. Thus, the methods to divide the tumor volume rationally and find a standard are yet inconclusive.

The differences in the cut-off points for tumor volume could be attributed to several reasons. The most common feature was the lack of consideration of individual tumor burden. The tumor burden could be embodied in several aspects, such as tumor volume. This phenomenon indicated that the same tumor volume could have different implications for different patients, i.e., different tumor burden which is crucial for the prognosis of patients.

In order to consider the individual tumor burden, we proposed the parameter of standardized tumor volume. SGTVnx was calculated by GTVnx/person’s volume and SGTVnd was calculated by GTVnd/person’s volume; both can reduce the impact of the individual tumor burden. Moreover, the ROC curve analysis determined the cutoff points, which were 1.091 and 0.273, respectively. The patients were categorized into 2 groups: SGTVnx > 1.091 *vs*. SGTVnx ≤ 1.091. The survival curves showed that patients with smaller SGTVnx had a better prognosis than those with larger SGTVnx, and the phenomenon was correlated with 5-year OS, DFS, DMFS, and LRFS rates in univariate and multivariate analyses.

Similarly, in SGTVnd, the survival curves also indicated that patients with SGTVnd ≤ 0.273 had higher 5-year OS, DFS, and DMFS rates as compared to those with SGTVnd > 0.273. Thus, SGTVnd was not established as an independent prognostic factor for the 5-year LRFS, due to the same factor presented by GTVnd.

In this study, we indicated that the tumor volume is an independent prognostic factor. Thus, we proposed the concept of standardized tumor volume and substantiated its effect on the prediction of prognosis in patients with NPC. In addition, a new pathway is also provided in order to divide the tumor volume rationally.

Nevertheless, there are some limitations in this study. Although we provide a new pathway to find the standard that can divide the tumor volume, additional studies with large sample size and unit participation are imperative.

In conclusion, standardized tumor volume is an independent prognostic factor for NPC. It can provide a new pathway to optimize the adequate application of the tumor volume.

The application of tumor volume might reduce the differences among different studies and add a new stage of classification.

## MATERIALS AND METHODS

### Patients

Between Jan 1, 2009 and December 30, 2012, 143 patients with a diagnosis of NPC based on histopathology were enrolled in the present study. The inclusion criteria were as follows: (1) UICC stage III–IVb (according to the 7th Union for International Cancer Control and American Joint Committee on Cancer), (2) age ≤ 70-year-old, (3) Karnofsky score > 70 points, (4) routine blood, liver, and kidney function tests were normal before the treatment, (5) absence of any history of previous radiotherapy, chemotherapy, or surgery (except diagnostic) of the primary tumor or node, previous or synchronous malignancy and complications, (6) received concurrent chemoradiotherapy, (7) underwent regular follow-up.

### Pretreatment evaluation

The essential pretreatment assessments included complete medical history, physical examination, nasopharyngeal fiberoptic endoscopy, MRI of the head and neck, chest radiography or CT, abdominal region ultrasonography or CT, bone emission CT scans, hematological and biochemical profile, and oral check. All the patients were required to provide written informed consents.

### Tumor volume definition

IMRT was performed in all patients with a linear accelerator (clinic IX, Varian, Palo Alto, CA, USA) using 6 MV photons. All patients underwent CT while immobilized in a supine position with a head–neck–shoulder thermoplastic mask, for the planning of radiotherapy. The whole region from the vertex cranii to2 cm below the clavicle head was scanned, with 3 mm slice thickness. The CT images were transferred to the planning system. The basis of the delineated tumor infiltration and the image of MRI, nasopharyngeal fiberoptic endoscopy, the treatment plan, and target of the tumor were delineated by two radiation oncologists; another radiologist was consulted in the event of a disagreement. The tumor volume can be automatically reconstructed to a three-dimensional image and calculated by the planning system in all patients. We reported the primary gross volume for nasopharynx (GTVnx) and the primary gross volume for lymph nodes (GTVnd). The primary gross volume for retropharyngeal lymph nodes was incorporated in GTVnx.

### IMRT replanning

GTVnx and GTVnd included the entire macroscopic tumor defined after correlative analysis by CT and MRI scans. For the clinical target, high-risk clinical target volume (CTV1) was defined as the nasopharynx gross tumor volume plus a 5–10 mm margin (2–3 mm posteriorly if adjacent to the brainstem or spinal cord) that can encompass the high-risk sites of microscopic extension and the whole nasopharynx. The low-risk clinical target volume (CTV2) was defined as the nasopharynx gross tumor volume plus a 5–10 mm margin (2–3 mm posteriorly if adjacent to the brainstem or spinal cord) that can encompass the low-risk sites of microscopic extension, including skull base, clivus, sphenoid sinus, parapharyngeal space, pterygoid fossae, posterior parts of the nasal cavity, pterygopalatine fossae, retropharyngeal nodal regions, and cervical nodes level II, III, IV, V. The total doses were prescribed to the median of the target volume, and the 95% isodose was approximately similar to the planning target volume (PTV). PTVnx, PTVnd, PTV1, and PTV2 were generated by adding 5mm margins to GTVnx, GTVnd, CTV1, and CTV2, respectively. The prescribed doses delivered to PTVnx, PTVnd, PTV1, and PTV2 were 68–72, 66–70, 60–64, and 52–56 Gy, respectively, in 30–32 fractions.

### Standardized tumor volume definition and calculation

We used the tumor volume divided by the person’s volume to obtain a relative tumor volume, which is defined as the standardized tumor volume.

Standardized primary gross volume for nasopharynx (SGTVnx) = GTVnx/person’s volume. Standardized primary gross volume for lymph nodes (SGTVnd) = GTVnd/person’s volume. Person’s volume = 1.015W-4.937 (W is person’s weight before treatment) [22].

### Chemotherapy

All patients underwent concurrent chemotherapy with cisplatin alone (80–100 mg/m^2^/day1/3 weeks). The chemotherapy was not delayed or paused until the nadir values were 1500 cells/μL or higher for neutrophils and 100,000 cells/μL or higher for platelets; the renal and liver functions were regained.

### Follow-up

During the treatment, all patients underwent weekly examinations, including nasopharyngeal fiberoptic endoscopy and the evaluation of the hematological and biochemical profile. Then, the patients were evaluated for tumor response through nasopharyngeal fiberoptic endoscopy and MRI. Subsequently, the patients were followed up every 3 months during the first 2 years, every 6 months for the third year, and every 1 years thereafter. Each follow-up included a physical examination, nasopharyngeal fiberoptic endoscopy, chest radiography or CT, abdominal region ultrasonography or CT, and hematological and biochemical profile. The MRI scan of the head and neck was performed every 6 months.

Additional CT and bone emission CT scans were performed to confirm any suspicious lesions for distant metastasis.

### Statistical analyses

ROC curve analysis was employed to confirm the cutoff point for GTVnx, GTVnd, SGTVnx, and SGTVnd. All the data were analyzed using SPSS17.0 statistical software. The overall survival (OS) was calculated from the date of diagnosis until death or the last follow-up using the Kaplan–Meier method, and the difference was analyzed using a two-sided log-rank test. The DFS, LRFS, and DMFS were also calculated and constructed using the Kaplan–Meier method. A univariate analysis was performed via the log-rank test and multivariate analysis through Cox regression. *P <* 0.05 was considered statistically significant.

## Abbreviations

NPC: Nasopharyngeal carcinoma
IMRT: Intensity-modulated radiotherapy
OS: Overall survival
HR: Hazard ratio
DFS: Disease-free survival
LRFS: Local relapse-free survival
DMFS: Distant metastasis-free survival
ROC: Receiver operating characteristic
